# HRCT imaging of acquired cholesteatoma: a pictorial review

**DOI:** 10.1186/s13244-019-0782-y

**Published:** 2019-10-03

**Authors:** Malvika Gulati, Swati Gupta, Anjali Prakash, Anju Garg, Rashmi Dixit

**Affiliations:** Department of Radiodiagnosis, Maulana Azad Medical College, Bahadur Shah Zafar Marg, New Delhi, 110002 India

**Keywords:** Cholesteatoma, Complication, High-resolution computed tomography, Mastoidectomy, Facial nerve

## Abstract

Chronically discharging ear is a common cause of morbidity in developing countries, and it is also associated with intratemporal and intracranial complications. The surgeon is often able to detect the disease. However, cholesteatoma in the “hidden areas” like anterior epitympanic recess and sinus tympani can be missed. Facial nerve involvement and cholesteatomatous erosion of the bony labyrinth are dreaded complications, the extent of which cannot be assessed completely on clinical examination. Adding to the complexity are the various variations in anatomy like high riding jugular bulb and aberrant internal carotid artery which could lead to catastrophic complications during surgery if left undetected preoperatively. HRCT temporal bone is useful to detect the extent of the disease, various complications, and guide the surgeon for pre-operative planning. In this review, we go through the various HRCT imaging features of acquired cholesteatoma, a reporting template, and a few words about imaging of the post-operative ear.

## Key points

- Acquired cholesteatoma is commonly associated with intratemporal and intracranial complications.
- Various “hidden areas” in the middle ear that are not amenable to clinical examination can be easily seen with HRCT.
- The extent of disease and complications can be assessed.
- Variant anatomy can be looked for to avoid intra-operative complications.

## Introduction

Cholesteatoma is a condition prevalent around the world, predominantly in developing countries. Acquired cholesteatoma is a common complication of unsafe/atticoantral chronic otitis media. Literally, it means the presence of “skin in the wrong place.” The cholesteatomatous sac is lined by stratified squamous epithelium constituting the matrix, which secretes the acellular keratin debris within [1]. An outer perimatrix contains mesenchymal cells which produce proteolytic bone destroying enzymes [2, 3].

Cholesteatoma can be classified as congenital or acquired. Another system of categorization could be according to the location of the cholesteatoma, which could be in the external auditory canal or middle ear cavity and other pneumatised parts of the temporal bone. HRCT temporal bone is currently advocated in all cases suspected of cholesteatoma clinically prior to surgery for assessment of the extent of disease, complications, and presence of variant anatomy.

## Theories regarding the formation of acquired cholesteatoma

Various theories exist regarding the formation of cholesteatoma; however, none of them explain it completely.

### Retraction pocket (invagination) theory

Being the most widely accepted theory, it states that the weakest part of the tympanic membrane (TM), i.e., pars flaccida, retracts because of the negative pressure in the middle ear cavity caused by long-standing eustachian tube dysfunction. This leads to the formation of posterosuperior attic pockets. The self-cleansing mechanism of these pockets is gradually lost with the accumulation of desquamation keratinized epithelial layer of the TM and results in the formation of keratin debris. Repeated superadded infection occurs and later biofilms are also formed over these debris which make them resistant to any antimicrobial therapy [4].

### Invasion

It is also hypothesized that there is an invasion of the keratinized squamous epithelium into the middle ear cavity through small perforations in pars tensa, which forms cholesteatoma.

### Metaplasia

Due to the chronic irritation, the keratinized squamous epithelium is formed from the squamous or cuboidal epithelium of the middle ear.

### Basal cell hyperplasia

There is a presence of microscopic defects even in the intact TM, through which epithelial cells can invade. These defects serve as direct contact between connective tissue of the mucoperiosteum with a middle ear effusion. In later stages, these areas become totally or partially covered with epithelium, and these epithelial breaks become connected to each other through the organized effusion. The cholesteatoma seems to spread by using the connective tissue as scaffolding [5].

Thus, acquired cholesteatomas can be classified based on the underlying pathogenesis as either “primary” or “secondary” where primary variety is seen behind pars flaccida of an intact retracted TM and the secondary variety grows into the middle ear through a perforated pars tensa part of TM [1].

The etiology of bony destruction by cholesteatomas is also debatable. Destruction only by mechanical pressure is unlikely. One of the theories states that production of degrading enzymes by osteoclasts is the cause of bony destruction [6, 7]. Due to its propensity to erode bone, the cholesteatoma can give rise to serious intracranial and extracranial complications.

## Clinical features

Clinically, the lesion presents with foul-smelling otorrhea, earache, and hearing loss. On otoscopic examination, it classically appears as a cluster of pearly white structures in the attic region. It is also suspected beneath polyps protruding from the pars flaccida or when there is a marginal TM perforation or granulation tissue [8].

Erosion of facial nerve canal can lead to features of lower motor neuron palsy manifesting as an inability to close ipsilateral eye, drooping of the corner of the mouth, and drooling of saliva. A large proportion of patients with facial canal erosion may not have features of facial nerve paresis. A labyrinthine fistula will lead to vertigo and nystagmus; however, these symptoms may be present in the absence of a fistula, often due to the diffusion of toxic substances into the intact labyrinthine. Intracranial complications can be life-threatening, patients presenting with fever, headache, and nuchal rigidity due to meningitis. The most common etiology of the cerebellar abscess is otogenic and patients present with fever and ataxia and other balance abnormalities.

## Technique of HRCT study

Non-contrast axial scans parallel to the axis of and through the petrous temporal bone are acquired on a multidetector CT scanner. Axial scanning is done with helical technique (120 kVp, high-resolution matrix (512 × 512), section thickness 0.6–1 mm, FOV 15–20 cm) in bone algorithm. Head is kept in neutral position and true axial images parallel to Reid’s line (joining inferior margin of the orbit to the center of the orifice of EAC) are acquired. Coronal multiplanar reconstruction is made perpendicular to the axial scans.

## Types of cholesteatoma

Cholesteatoma can be congenital or acquired. *Congenital cholesteatoma* of the temporal bone is formed due to lack of involution of distinct squamous cell rest, which should have involuted to become normal endothelium [1]. Middle ear cavity including mastoid, petrous, and squamous portion of the temporal bone and TM are frequent sites of congenital cholesteatoma. Another location is intradural (cisternal) usually involving the cerebellopontine angle (CPA) and the patient presents with varying degrees of hearing loss [9, 10]. A solitary lytic calvarial lesion may be cholesteatoma in the diploic space. They are usually incidental findings on imaging and history of ear discharge or previous otologic procedure is lacking. Usually, the TM is intact and retraction pockets are not seen in such cases. Presence of cholesteatoma in the anterosuperior part of the middle ear cavity just above the opening of the eustachian tube should raise the suspicion of congenital etiology [2].

The other type of cholesteatoma is the *acquired* variety. The acquired variety can be further classified according to the location of the cholesteatoma in the external or the middle ear.

*EAC cholesteatoma* is uncommon and usually occurs in an age group older than middle ear cholesteatoma. It appears as a soft tissue mass in the canal particularly along its floor with associated bony destruction and periostitis. It occurs due to the invasion of the squamous epithelium lining the EAC into the underlying bone. However, it is difficult to distinguish between cholesteatoma, carcinoma, and malignant otitis externa using HRCT alone [2, 11].

**Fig. 1:**
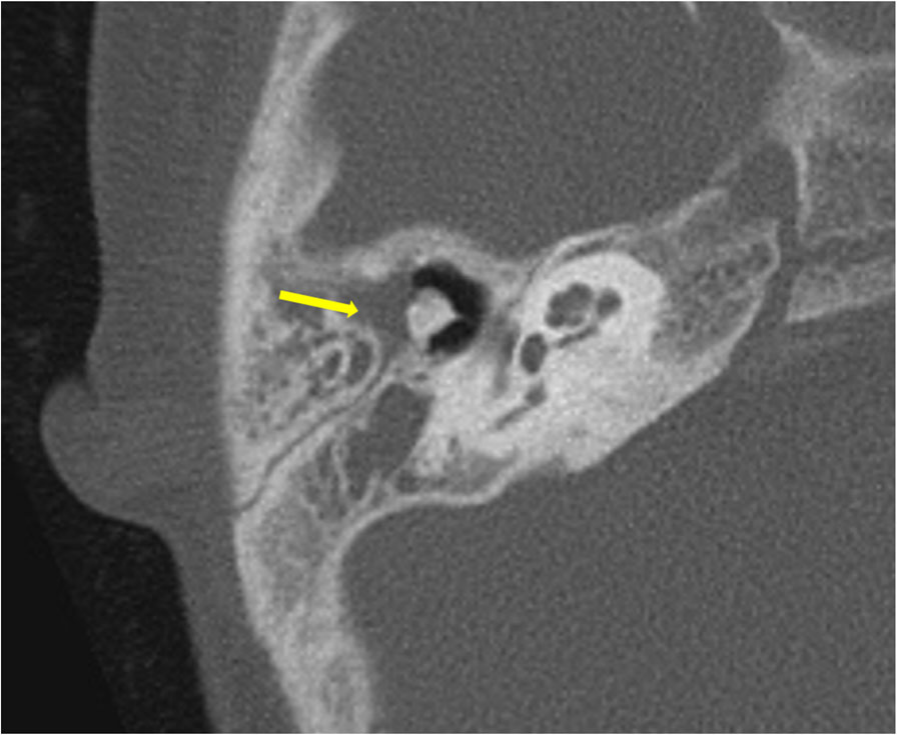
Axial CT image showing non-dependent soft tissue in the Prussack’s space, lateral to ossicles in a patient with pars flaccida cholesteatoma (arrow)

**Fig. 2:**
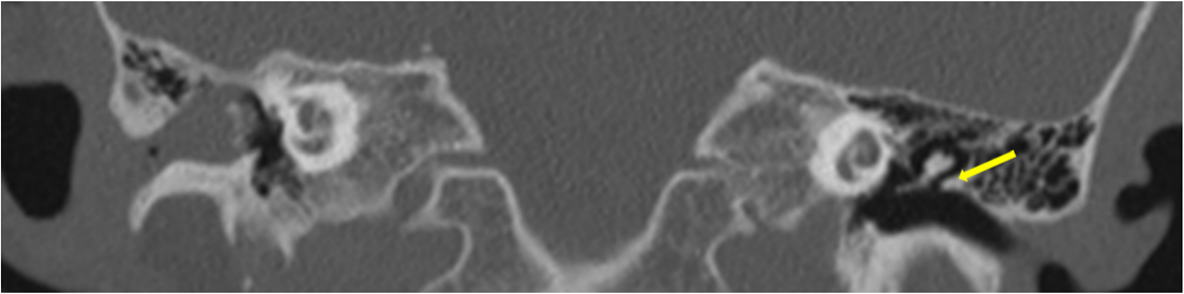
Coronal CT image showing normal scutum on the left side (arrow). It is seen as a point of superior attachment of the tympanic membrane. Scutum is eroded on the right side with soft tissue seen in the middle ear and external auditory canal

**Fig. 3:**
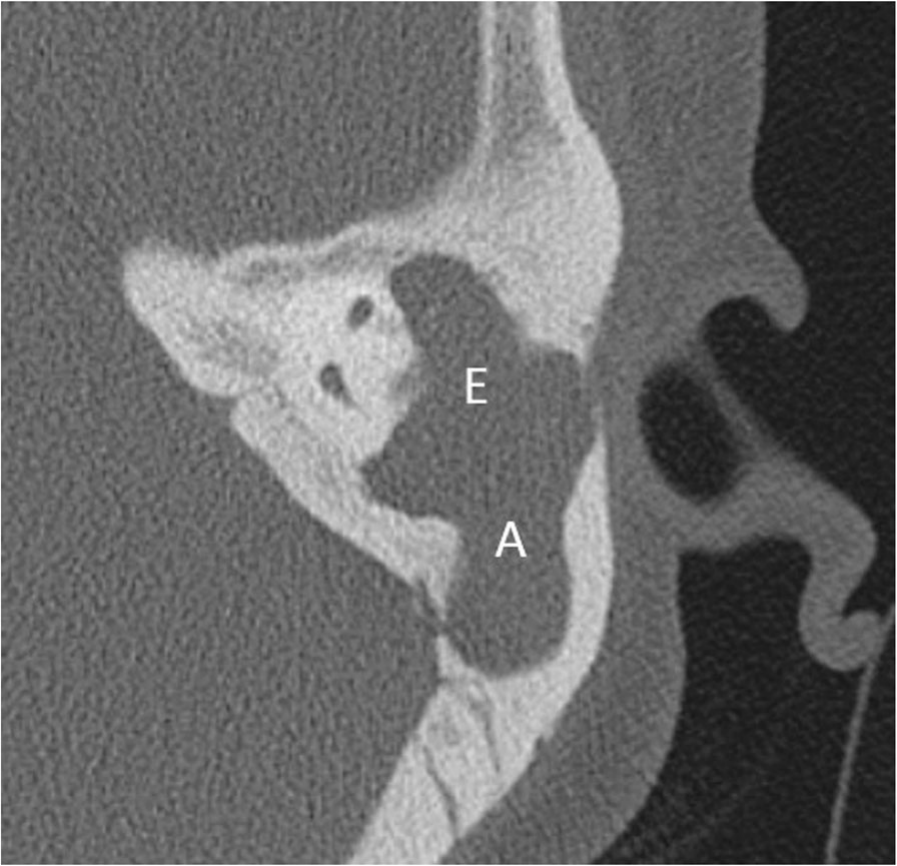
Axial CT reveals widening of aditus (denoted by A) and formation of common cavity between epitympanum (denoted by E) and aditus with soft tissue within

Among the *middle ear cholesteatoma*, the most common type is *pars flaccida* variety and is seen characteristically in the Prussack’s space, a niche in the epitympanum lateral to the ossicles. These cause medial displacement of the malleus and erosion of the bony scutum, extending into the aditus and antrum posteriorly (Figs. 1 and 2). Widening of the aditus and formation of a common cavity are a characteristic feature [12, 13] (Fig. 3).

**Fig. 4:**
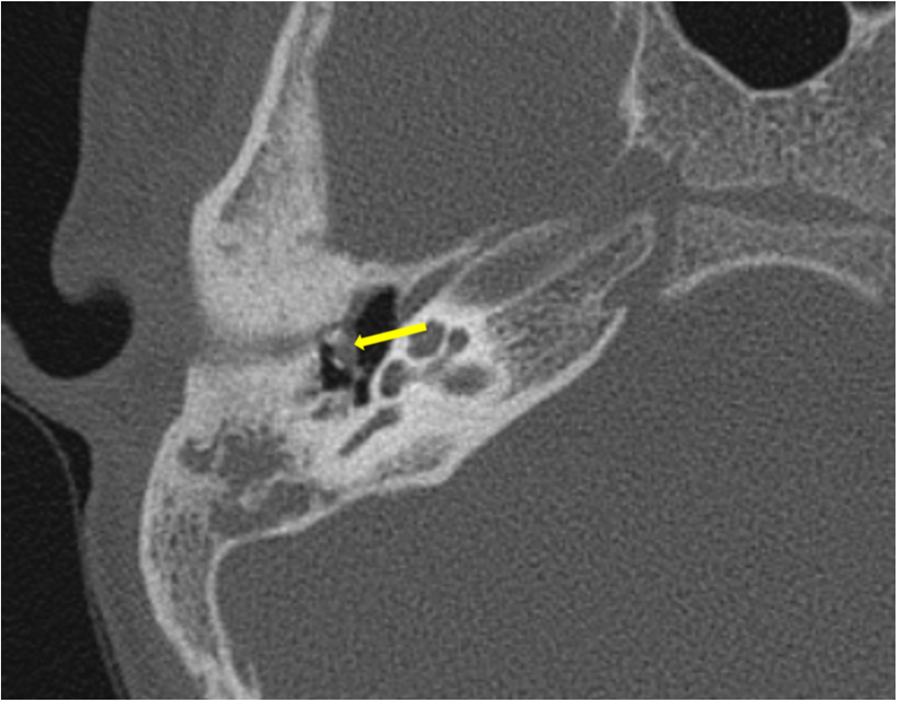
Axial CT image showing non-dependent soft tissue medial to ossicles in a patient with pars tensa cholesteatoma (arrow)

**Fig. 5:**
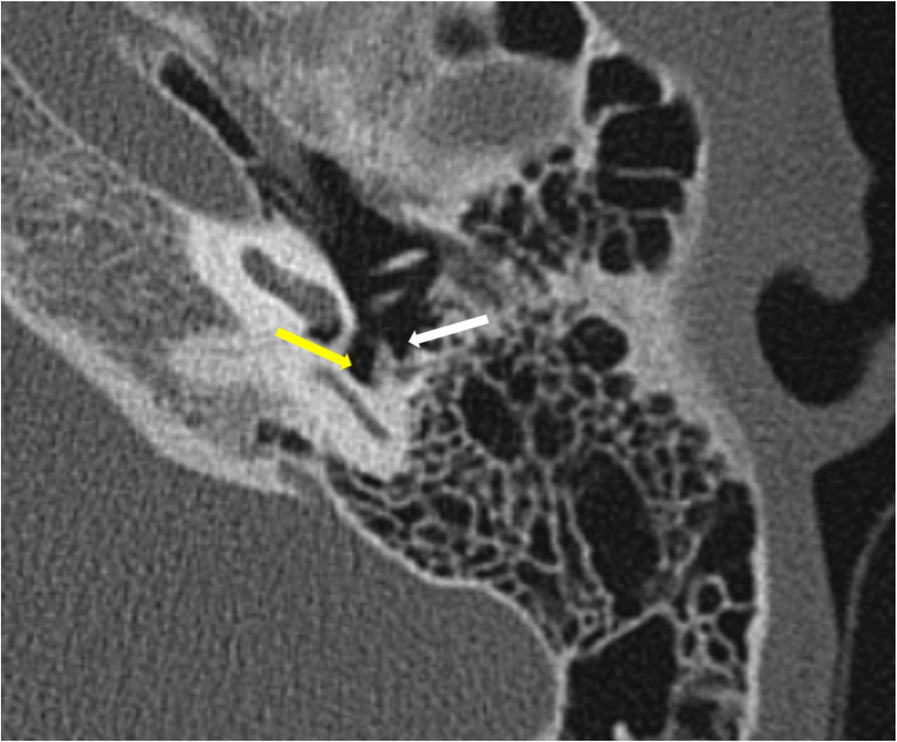
Axial CT through the mesotympanum shows the sinus tympani (yellow arrow) and facial recess (white arrow) which are hidden areas by otological examination and should be carefully inspected on imaging for the presence of soft tissue.

**Fig. 6:**
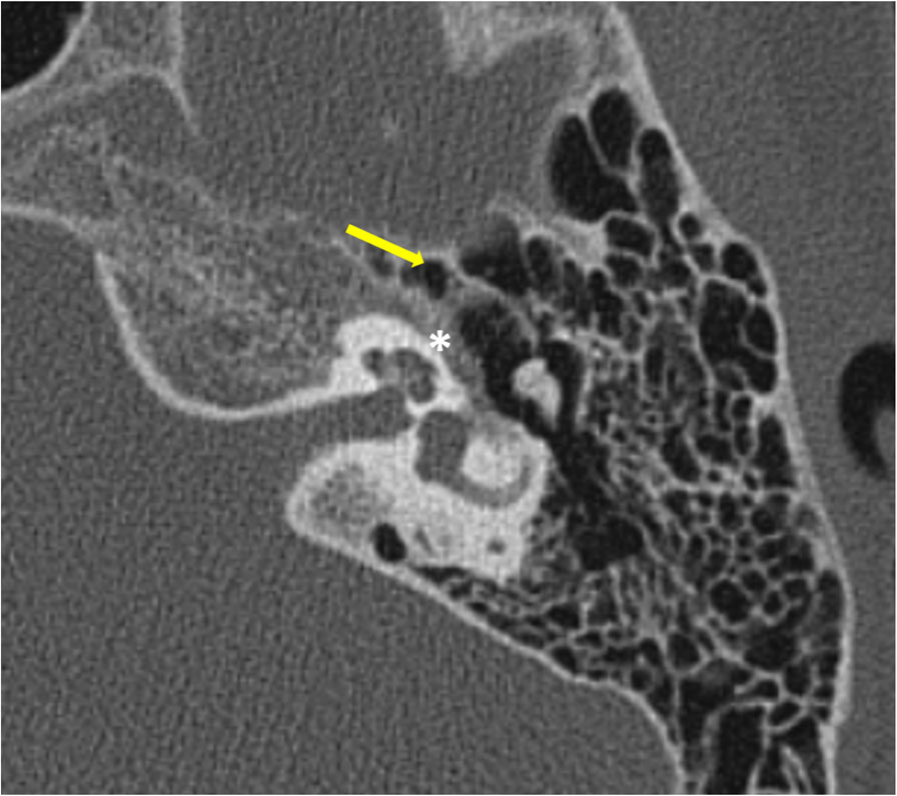
Axial section through the epitympanum shows the normal anterior epitympanic recess (arrow) which is the medial extension of epitympanum. Its importance lies in its close proximity to the tympanic segment of the facial nerve canal (*)

*Pars tensa* variety is less common and usually arise within retraction pockets. Pars tensa cholesteatoma displaces the ossicles laterally in contrast to pars flaccida variety [9] (Fig. 4). Careful inspection of *sinus tympani and facial recess* is necessary as these areas are not visible on otoscopic examination (Fig. 5). Another important location in the middle ear is *anterior epitympanic recess* also called supratubal recess, which is the medial most extension of the epitympanum. A bony bar called cog which is attached to the tegmen anterior to the malleus forms the boundary between anterior epitympanic recess and epitympanic cavity**.** Medially, this recess is in close relation to the proximal part of tympanic segment of the facial nerve and geniculate ganglion, and dehiscence of the bony partition can make the facial nerve prone to injury [14, 15] (Fig. 6)

## Imaging features of cholesteatoma

The hallmark of cholesteatoma is bony destruction. Presence of soft tissue density in the middle ear cavity coexistent with ossicular and mastoid bony erosion is highly specific for cholesteatoma. In attic cholesteatomas, the ingrowth of epithelium from the pars flaccida into the epitympanum is evident by the erosion of the scutum.

**Fig. 7:**
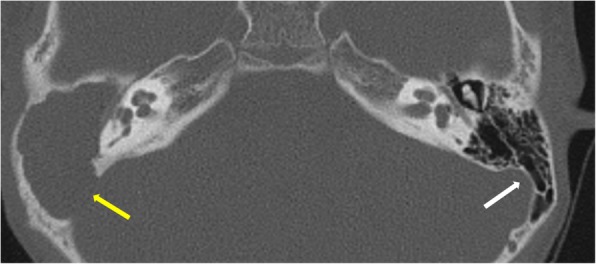
Axial CT shows normal “ice cream cone” configuration of ossicles in epitympanum on the left side formed by the head of the malleus and the body of the incus. The right ear shows soft tissue in the middle ear and aditus with non-visualization of ice cream cone suggesting erosion. A large defect is also seen in the sinodural plate on the right side (yellow arrow). Left sinodural plate is intact (white arrow)

**Fig. 8:**
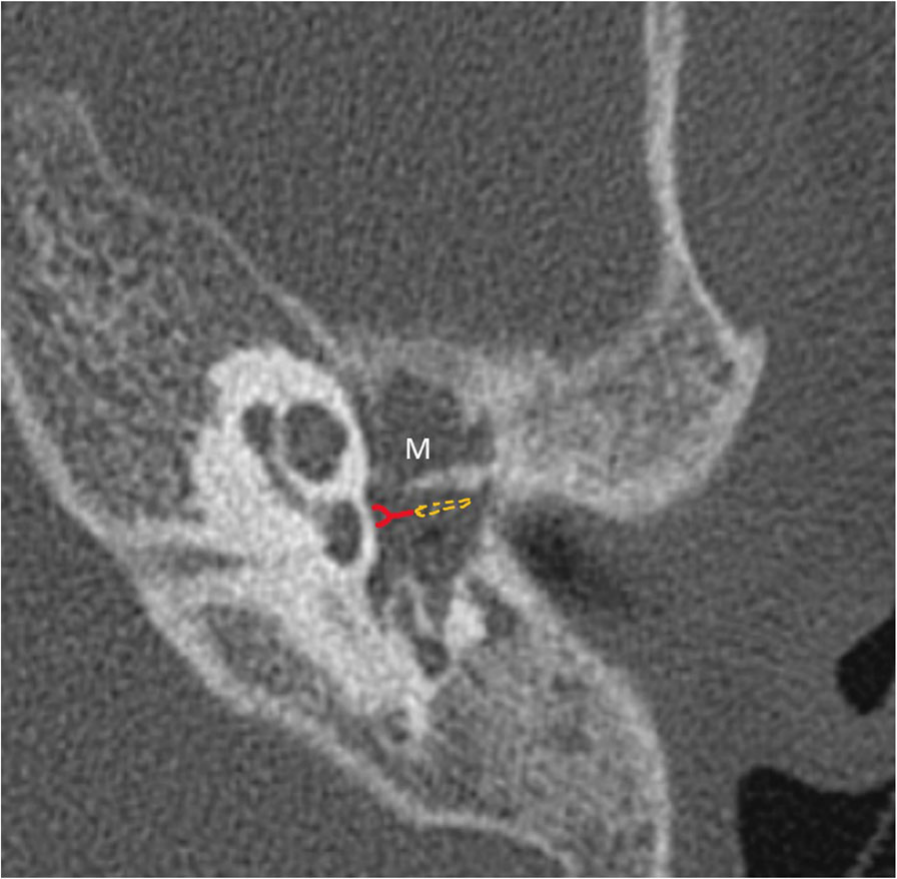
Axial CT shows soft tissue in the mesotympanum (M) with erosion of long process of incus and stapes which are not visualized in their expected locations. The line diagram illustrates the normal location of stapes (red) and long process of incus (yellow).

Ossicular erosion is more frequently encountered in pars tensa cholesteatoma than pars flaccida location of soft tissue. Ossicles may not be visualized, appear rarefied, or show small erosions. Lenticular process of the incus is most prone to erosion, due to poor ligamentous support and precarious arterial supply by end arteries. Disruption of the “ice cream cone” configuration of ossicles in epitympanum is seen frequently in attic cholesteatoma where the “ice cream scoop” is the head of the malleus and the body of the incus represents “the cone” (Figs. 7 and 8). Careful inspection of the footplate of stapes at oval window niche is also particularly important. Although the degree of ossicular erosion does not alter the need for surgery, it can help prognosticate the patient regarding recovery of hearing loss after surgery and aids in planning the reconstructive procedure. For instance, patients showing erosion of stapes superstructure experience a much higher degree of hearing loss compared to patients with intactness of the same. Correlation for ossicular erosion between CT and surgical findings is higher for the malleus, body, and short process of incus when compared to the long process of incus and stapes.

However, in the absence of bony erosions, diagnosis of cholesteatomas is challenging on CT. Presence of non-dependant soft tissue goes in favor of cholesteatoma. Also, mass effect on the ossicles is frequently seen in cholesteatomas, even in the absence of frank destruction [2].

**Fig. 9:**
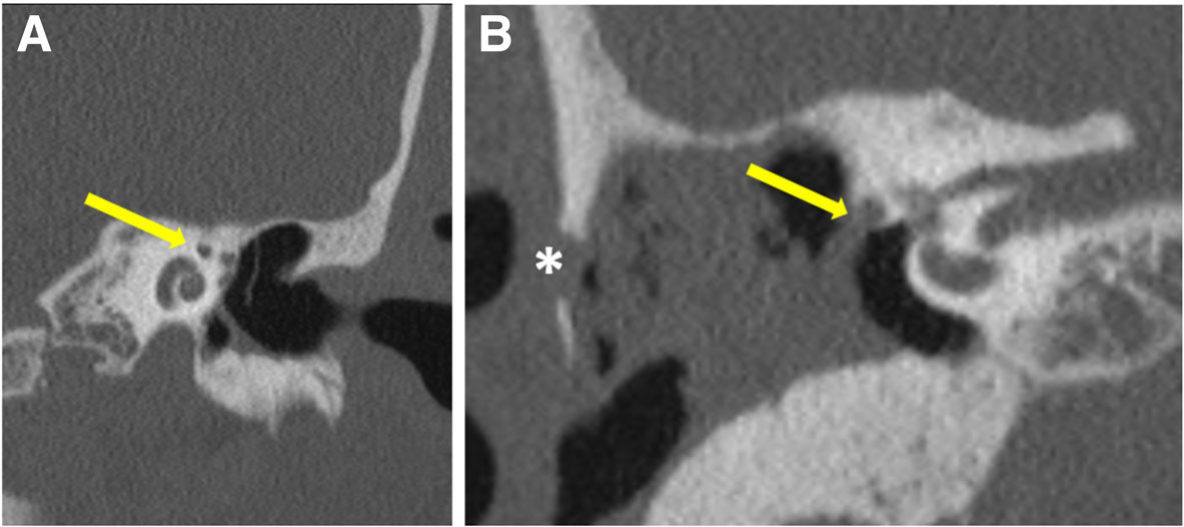
Coronal (**a**) CT image reveals the “snail eye” view where the turns of cochlea form the body of snail and labyrinthine and tympanic segments of facial nerve being the eyes (arrow). Coronal (**b**) CT image shows the erosion of the floor of the horizontal segment of the facial canal (arrow). Erosion of lateral mastoid cortex is also seen (asterisk)

**Fig. 10:**
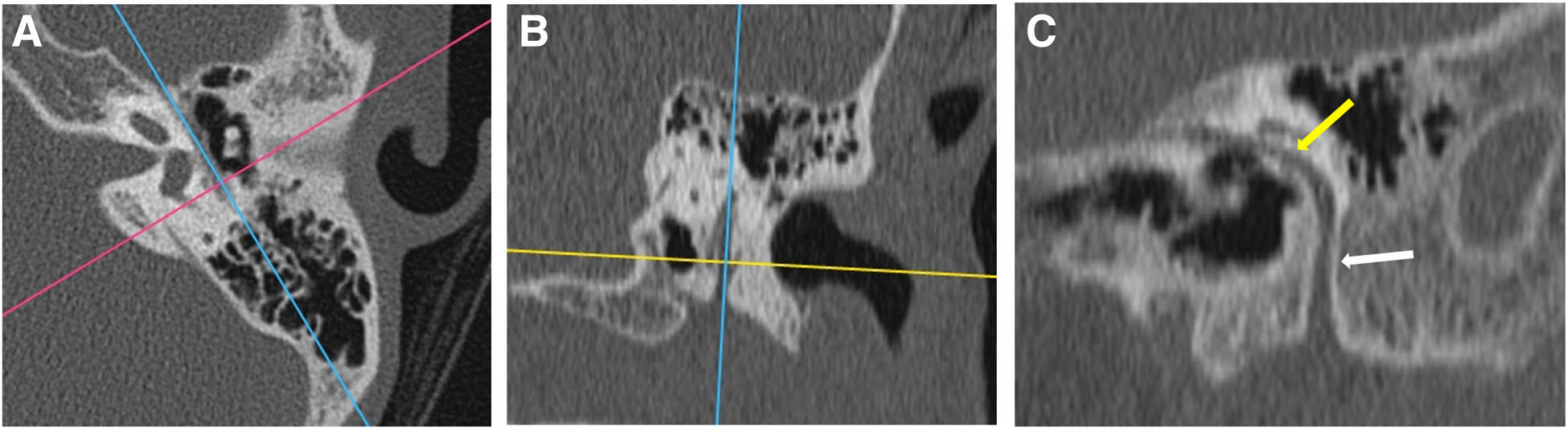
Axial (**a**) and coronal (**b**) reference CT images show plane of reconstruction (blue line) to form the double oblique sagittal (**c**) image showing normal anatomy of tympanic (yellow arrow) and mastoid segments (white arrow) of the facial nerve canal

*Erosion of the facial nerve canal* is an important complication. Tympanic/horizontal segment is the most frequently affected. A limitation exists in evaluating the facial canal as it can be so thin even in the non-pathological ear that it becomes difficult to assess the presence of its erosion in the affected ear. This explains for the poor radiological correlation with the surgical findings [16, 17]. In addition, visualizing the tympanic portion of the facial canal is found to be especially difficult when there is an adjacent pathologic soft tissue in the mesotympanum [8]. Routinely, axial and coronal reconstructions are seen to evaluate the facial canal (Fig. 9a, b). Double oblique sagittal images, along the plane of tympanic segment, aid in better visualization of this segment (Fig. 10).

**Fig. 11:**
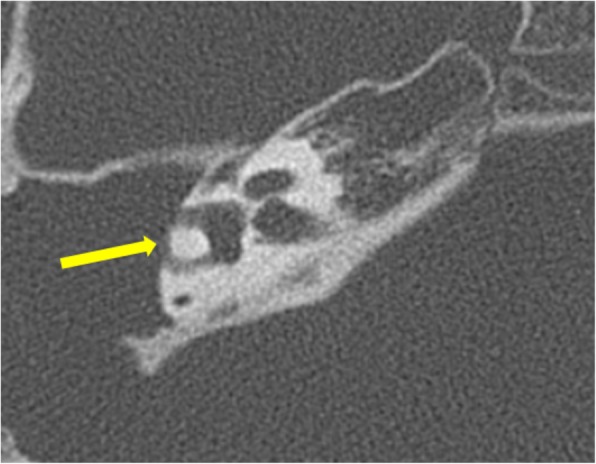
Axial CT image showing erosion of the lateral wall of the lateral semicircular canal (arrow) with the formation of labyrinthine fistula

*Labyrinthine fistula* is a dreaded complication of cholesteatoma. The most commonly affected is the lateral semi-circular canal due to its close proximity to the middle ear cavity (Fig. 11). Extensive disease can affect cochlea and other semi-circular canals also. Fistula formation is seen as direct contact between the soft tissue and membranous labyrinth. Hence, detailed inspection of the labyrinth is necessary in axial and coronal planes. Even then, very small fistulae can be missed. The presence of cholesteatoma in close proximity to the bony labyrinth also needs to be reported even in the presence of intact intervening bone, due to the danger to the underlying labyrinth during surgery [18].

*Erosion of lateral mastoid cortex* is also commonly seen (Fig. 9b).

**Fig. 12:**
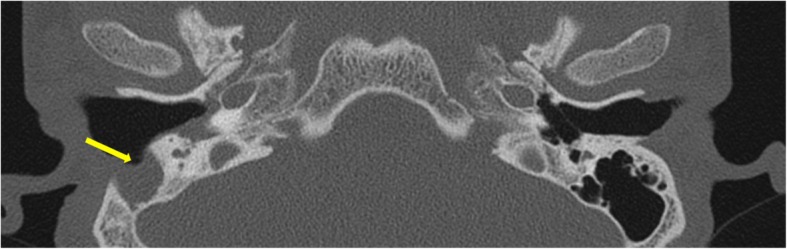
Axial CT shows the right mastoid cavity filled with soft tissue and is seen to open directly into the external auditory canal suggesting automastoidectomy (arrow)

Extensive bony destruction of the mastoid and ossicles can occur which may give the appearance similar to postsurgical cases termed as *automastoidectomy*. Spontaneous evacuation of cholesteatoma may be seen with this, termed as *mural cholesteatoma* leaving behind no residual mass [2] (Fig. 12).

**Fig. 13:**
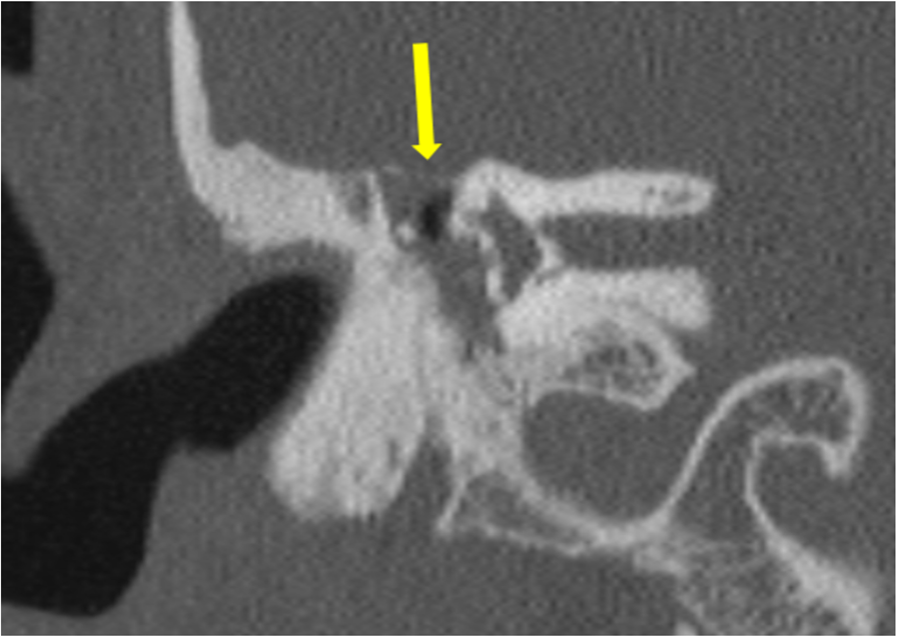
Coronal CT image showing erosion with focal defects in tegmen tympani (arrow).

Other sites of erosion include *tegmen tympani* (Fig. 13) and *sinodural plate* (Fig. 7). Erosion of the sinodural plate is known to be a common implicating cause of transverse sinus thrombosis, meningitis, cerebritis, and abscess. In suspected intracranial complications, MRI is the investigation of choice.

## Differential diagnosis

The differential diagnosis of *soft tissue in the middle ear with bony erosions* includes cholesteatoma, facial nerve schwannoma/hemangioma, paraganglioma, rhabdomyosarcoma, LCH (Langerhans cell histiocytosis), squamous cell carcinoma, metastasis, and GCT (giant cell tumor) [1]. The presence of retraction of tympanic membrane points towards chronic suppurative otitis media as the etiology [18].

HRCT is unreliable in differentiating residual or recurrent cholesteatoma from other entities which usually occur *without bone destruction*, like granulation tissue, cholesterol granuloma, post-inflammatory ossicular fixation, mucosal edema, fibrosis, scar tissue, and fluid. They are also frequently encountered in the post-operative period. MR imaging can usually aid in making the correct diagnosis as cholesteatomas are hypointense on T1WI, hyperintense on T2WI, do not show any enhancement, and demonstrate diffusion restriction on DWI. Cholesterol granulomas on the other hand show hyperintense signal on T1WI due to the presence of intrinsic blood products [2]. Post-inflammatory ossicular fixation is a complication of non-cholesteatomatous chronic otitis media and appears as soft tissue density encasing the ossicles particularly in the oval window niche with or without calcific foci. Absence of ear discharge and the presence of calcification or fibro-osseous tissue are pointers towards non-cholesteatomatous etiology [18].

### Preoperative assessment

In addition to the evaluation of complications of cholesteatoma, imaging also helps in warning the surgeon of any variant anatomy that may predispose to life-threatening consequences during surgery or may affect surgical access to disease such as the following:

- Dehiscent facial canal
- High and dehiscent jugular bulb
- Anteriorly lying sigmoid sinus
- Low lying tegmen and dura [8]
- Specific patterns of pneumatization and aeration of mastoid [19].

### Imaging of post-operative ear

Imaging is of particular importance in patients who have undergone canal wall up procedures where rates of recurrence range from 18–36%, and detection of residual or recurrent disease by otoscopic means is particularly tough [20].

In the postoperative period, HRCT has a high negative predictive value in cases with no evidence of soft tissue densities. However, bony erosion in patients who have previously undergone a tympanomastoidectomy cannot be characterized, as it is impossible to differentiate between surgical changes and pathological bony destruction due to cholesteatoma. In this setting, HRCT has a sensitivity of 43%, specificity of 42–51%, and a predictive value of 28% for detection of residual or recurrent cholesteatoma. HRCT also plays a major role prior to revision surgery, as anatomy may be considerably altered, limiting the utility of normal surgical landmarks [19].

If an HRCT shows no abnormal soft tissue at 6 or 9 months following the initial surgical procedure, the surgeon may be more confident in delaying a second-look surgery [19].

## Limitations of HRCT

HRCT temporal bone has certain limitations. It is difficult to differentiate cholesteatoma from granulation tissue, pus, or fluid. Early disease with the absence of osseous erosion is challenging to pick. The integrity of the facial canal cannot be accurately commented upon, especially in the presence of adjacent soft tissue in the middle ear. Due to the acquisition of the non-contrast scan, intracranial complications are frequently overlooked.

## Checklist for acquired cholesteatoma on CT

- Location of mass—epitympanum/mesotympanum/hypotympanum
- Hidden areas—sinus tympani, facial recess, anterior epitympanum recess
- Ossicles
- Osseous walls of the middle ear
- Mastoid air cells, aditus
- Tegmen tympani, sinodural plate, scutum
- Facial canal
- External auditory canal and tympanic membrane
- Inner ear esp. lateral semi-circular canal
- Petrous apex, intracranial abnormality, post-auricular soft tissue
- Variant anatomy

## Conclusions

HRCT temporal bone offers an insight into the extent of cholesteatoma and the various complications before the surgeon embarks onto the definitive line of management. It also helps detects recurrent/residual middle ear disease to avoid unnecessary second-look surgery.

## Data Availability

Not applicable

## Abbreviations

CPA: Cerebellopontine angle
DWI: Diffusion-weighted imaging
EAC: External auditory canal
esp.: Especially
FOV: Field of view
GCT: Giant cell tumor
HRCT: High-resolution computed tomography
KVp: Kilovolt peak
LCH: Langerhans cell histiocytosis
MR: Magnetic resonance
TM: Tympanic membrane
WI: Weighted images
